# The Effect of Young People–Assisted, Individualized, Motion-Based Video Games on Physical, Cognitive, and Social Frailty Among Community-Dwelling Older Adults With Frailty: Randomized Controlled Trial

**DOI:** 10.2196/57352

**Published:** 2024-11-20

**Authors:** Arkers Kwan Ching Wong, Melissa Qian Zhang, Jonathan Bayuo, Karen Kit Sum Chow, Siu Man Wong, Bonnie Po Wong, Bob Chung Man Liu, David Chi Ho Lau, Tobias Kowatsch

**Affiliations:** 1 School of Nursing, The Hong Kong Polytechnic University, Hung Hom, China (Hong Kong); 2 Hong Kong Lutheran Social Service, Homantin, China (Hong Kong); 3 Step Health, Sheung Wan, China (Hong Kong); 4 School of Medicine, University of St.Gallen, St Gallen, Zurich, Switzerland; 5 Institute for Implementation Science in Health Care, University of Zurich, Zurich, Switzerland; 6 Centre for Digital Health Interventions, Department of Management, Technology, and Economics, Eidgenössische Technische Hochschule Zürich, Zurich, Switzerland

**Keywords:** frailty, gaming intervention, motion-based, video games, older adults, gerontology, geriatrics, randomized controlled trial, RCT, physical fitness, adolescents, young people–assisted, eHealth literacy, well-being, therapists, youth volunteers, social support, exergames, gamification, active games, physical activity

## Abstract

**Background:**

The aging population highlights the need to maintain both physical and psychological well-being. Frailty, a multidimensional syndrome, increases vulnerability to adverse outcomes. Although physical exercise is effective, adherence among older adults with frailty is often low due to barriers. Motion-based video games (MBVGs) may enhance motivation and engagement.

**Objective:**

This study aims to evaluate the effect of individualized exercise programs that combine MBVGs, intergenerational support, and therapeutic frameworks on physical, cognitive, and social frailty outcomes in community-dwelling older adults.

**Methods:**

This randomized controlled trial was conducted from March 2022 to October 2023 across 6 community centers in Hong Kong. Participants aged 60 years and above with mild neurocognitive disorder were recruited, screened, and randomly assigned to either an intervention (n=101) or control group (n=101). The intervention included an 18-week program with 12 supervised exercise sessions utilizing motion-based technology, led by occupational therapists and assisted by youth volunteers. Data were collected at baseline (T1) and postintervention (T2), focusing on physical, cognitive, and social frailty outcomes, as well as client-related metrics. Statistical analyses were performed using SPSS, with significance set at *P*<.05.

**Results:**

A total of 202 participants were recruited, with a mean age of 78.8 years (SD 7.8). Both groups showed improvements in balance from T1 to T2, with a significant time effect (*β*=−0.63, *P*=.03). The intervention group demonstrated enhancements in hand strength and BMI, but no statistically significant between-group differences were observed. The intervention group also exhibited significant improvements in cognitive function (*β*=2.43, *P*<.001), while the control group’s scores declined. Short-term memory improved for both groups, with no significant differences noted. Both groups experienced a reduction in depression levels, with a significant within-group effect at T2 (*β*=−1.16, *P*=.001). Improvements in social connectedness and eHealth literacy were observed in both groups, with the latter showing a significant within-group effect at T2 (*β*=3.56, *P*=.002). No significant effects were found for social isolation, physical activities, or quality of life.

**Conclusions:**

The growing aging population necessitates innovative strategies to support aging in place. Results indicated statistically significant improvements only in BMI and cognition, while other outcomes such as loneliness, balance, and eHealth literacy showed positive trends but lacked significance. Despite the limitations observed, particularly regarding the role of volunteer support and the diverse needs of community-dwelling older adults, the findings contribute to the foundation for future research aimed at enhancing biopsychosocial outcomes. Future studies should explore tailored interventions that consider individual preferences and abilities, as well as evaluate specific components of motion-based video games to optimize their effectiveness.

## Introduction

### Aging and Frailty

There has been a dramatic growth in the aging population over the past decade due to improved life expectancy. Although prolongation of life is one of the most significant goals of public health [1], maintaining both physical and psychological well-being at older ages is a complex and challenging issue [2]. Frailty is a geriatric syndrome that arises with increasing age, which is distinct from any single chronic illness [3]. Researchers have found that older adults with frailty are vulnerable to adverse outcomes including higher risk of falls, disability, mortality, hospitalization, and institutionalization [4-6]. Fried et al [7] developed a phenotypic description of frailty that consists of 3 or more of the clinical components: muscle weakness, low physical activity level, sense of low energy or exhaustion, unintended weight loss, and slowed walking speed. The Rockwood scale described frailty as the accumulation of deficits including cognitive impairment, functional deterioration, and number of diseases [8]. Current thinking regards frailty as a multidimensional concept, and focuses on not only physical but also psychological and social contributors for the definition and treatment of frailty [9]. Despite its varying definition, older adults with frailty typically are physically inactive, have less social integration, and have chronic diseases requiring medical and general care [6]. Frailty is a chronic and progressive process for 90% of those becoming frail [10]; thus, interventions to slow the progression of frailty and optimize health outcomes are crucial.

### Physical Exercise and Motion-Based Video Games

Numerous studies have shown that physical exercise is a promising, low-risk, and effective therapeutic approach to mitigating frailty [3,6]. Evidence suggests that regular physical exercise is associated with improved physical performance (eg, muscle strength, balance, endurance for activity) [11], decreased risk of cognitive impairment [12], and improved psychological well-being (eg, emotions, self-efficacy, life satisfaction) [13]. Although physical exercise is broadly recognized to bring health benefits, motivation and adherence to physical exercise are suboptimal among older adults with frailty. Aside from an individual’s age-related impairment, other barriers that limit exercise engagement include transportation restrictions, inclement weather, lack of space, and economic issues [2]. Adopting a creative intervention such as applying motion-based technology with exercise could be one alternative option to solve some of the motivational barriers to physical exercise. Motion-based video games (MBVGs), defined as “exergames” in some studies, are generally understood to combine video games or multimedia interactions with physical activities, which means players are required to perform specific physical movements to complete tasks assigned by the video game interface [14]. Due to the gamification features, game-based exercise is believed to enhance motivation and adherence since it provides real-time interaction as well as visual and audio performance feedback [15]. Moreover, this activity can be applied in any context, such as in home, community, and hospital settings [16].

Although the effectiveness of MBVGs in improving physical heath (eg, functional mobility, balance, gait performance) has been reported in a considerable number of studies [16,17], only a few studies have investigated their psychological impact on older adults as the main focus. As demonstrated in previous studies, positive psychological effects can result from frequent physical exercise [18]. Since older adults are less interested in proactively improving their health, better engagement and immersion in activities can be achieved when they view activities as being intrinsically attractive and enjoyable, and this can result in positive emotions [6]. The psychological effect should be regarded as an indicator of MBVG effectiveness in addition to the physical effect [19]. Several studies illustrated a positive effect of MBVGs on improving mental health for community-living older adults. A qualitative study provided a 10-week program involving a group play exergame for 16 older adults with serious mental illnesses; it found that group play through exergames elicited positive emotions and self-efficacy [20]. A study conducted by Kahlbaugh et al [21] randomly assigned 35 community-dwelling older participants to either play activity simulation games (bowling) with a young partner or watch television with a young partner for 10 weeks; decreased loneliness and greater positive mood were reported in the group who bowled. Another study compared the antidepressive effect between an exergame group and traditional exercise group among 102 older adults during a 6-week period, and the exergame group was found to have more positive emotions and less depressive symptoms [14]. In addition, participation in MBVGs has been recognized as a social activity that facilitates player interaction, thereby fostering the development of social connection and friendships among participants [15]. Studies have delved into the potential social benefits of MBVGs and found it can reduce the loneliness level of older adults, which may be attributed to increased social interaction and connectedness with other participants rather than the act of playing the game itself [22]. A study by Chao et al [16] suggested that the social benefits of MBVGs may also enhance exercise motivation and adherence among older adults.

So far, very little attention has been paid to the psychological and social effect of MBVGs on frail community-dwelling older adults. In addition, the majority of existing research has used commercially available motion-based technologies such as the Nintendo Wii (an interactive exercise video program) or Kinect (for the Xbox 360 game system) in the training process, which are not designed specifically for older people, especially those with frailty. However, an individualized program is necessary for people with frailty in order to prevent adverse outcomes, and extra care is required to reassure people to engage with the program. To encourage those with frailty to participate in the exercise program, the individual’s needs and abilities should be considered, as well as the best type of activity to reach their goals [23].

### Strategies for Individualized Programs

Frames of reference are commonly used guidelines for addressing the impairments that pose barriers to activity performance [24]. Older adults vary in the level of understanding they have about their health conditions and the impact that their current conditions have on their life experience. The Biomechanical Frame of Reference is primarily concerned with an individual’s capacity for movement (range of motion, muscle strength, and endurance) in the context of performing daily activities and is usually carried out by linking impairments to performance deficits [25]. This framework can be used to improve a person’s perception of their current health situation, highlighting how ongoing or chronic dysfunction affects their performance of daily activities. With respect to older adults with considerably irreversible dysfunction, a compensatory approach is necessary to enable those with impairments to regain independence in daily activities. The Compensatory Frame of Reference provides compensatory techniques for individuals who have experienced functional decline to reengage in activities, such as by using assistive devices to compensate for dysfunction in their desired occupations [26]. Other than applying frames of reference that focus on addressing specific impairments, the Person-Environment-Occupation (PEO) model is mainly utilized as a guide to organize the person, environment, and occupation factors to create complete person-centered intervention plans, which help to achieve the overall well-being of the older adults [25]. This conceptual model points out that the performance of activities can be optimized when the environment and the occupation are aligned to support activities [25]. Thus, the congruent environment plays an important role in maximizing a person’s quality of performance. The integration of the frames of reference and conceptual model is considered to assist in individualizing exercise programs through enhancing a person’s perception of their specific health conditions and their impact, applying assistive technology based on one’s functional level, and conducting training within a carefully assessed environment.

Intergenerational programs have been utilized widely; this refers to a process to bring together older adults and young people in a collaborative context. It is generally believed that young people are more adaptive to new conditions and capable of acquiring new technologies in a short period of time [27] compared to older adults. Older adults aged over 65 years are less likely to adopt new technologies, and often show decreased self-efficacy and performance while interacting with digital interfaces [28,29]. Barriers for older users of technology include a lack of experience, a lack of guidelines for this particular group, and age-related changes. A study by So and Shek [30] indicated that older adults have higher motivation and enthusiasm to learn new knowledge when working with young generations. Teater’s study [31] also found that intergenerational contact enhanced older adults’ sense of self-worth and social interaction. An intergenerational program is considered to be an approach that can equip older adults with the competence to engage in technology-based exercise with the guidance and support of young volunteers.

Previous studies broadly classify psychological outcome measures into 4 categories: emotions (eg, depression, anxiety), self-perceptions (eg, self-efficacy, self-concept), bodily well-being (eg, physical symptoms), and global well-being (eg, life satisfaction, overall well-being) [13]. An abundance of studies found a connection between exergames and improved psychological outcomes, including self-efficacy, life satisfaction, and depression [32]. Although some research has been carried out on the psychological impact of game-based exercise among older adults, there are few published randomized controlled trials that have investigated the effect of MBVGs on psychological outcomes for community-dwelling older adults, especially those with frailty. Furthermore, no single study exists that has combined frames of reference, a conceptual model, and intergenerational support into an individualized game-based exercise regime, which may be able to address the engagement and adherence issues that are encountered in traditional exercise programs. The aim of this study is to test the effect of individualized exercise programs, using the combination of frames of reference, intergenerational support, and MBVGs, on physical frailty outcomes (balance, handgrip strength, blood pressure, BMI), cognitive frailty outcomes (cognition, short-term memory), social frailty outcomes (loneliness, social isolation), and client outcomes (physical activities, quality of life, depression, self-efficacy, social connectedness, eHealth literacy) among frail community-dwelling older adults. The results might suggest a new approach to improve the holistic health of older adults with frailty, provide a comprehensive view on how to better address the needs of older adults, and guide a more effective use of resources to deliver physical as well as mental health services in the community.

## Methods

### Design

The study was a randomized controlled trial conducted between March 2022 and October 2023 at 6 community centers run by a nongovernmental organization in Hong Kong. The study protocol was approved by the Human Ethics Sub-committee of the Hong Kong Polytechnic University (HSEARS20220225001) and registered at ClinicalTrials.gov (NCT05267444).

### Participants and Recruitment

The community centers assisted in screening and contacting eligible participants from their 50,000 service users. Subjects who were members of the centers and who showed an interest in this program were screened and recruited into the study if they (1) lived in the community; (2) were aged 60 years or above; (3) had a mild neurocognitive disorder with a Hong Kong version of the Montreal Cognitive Assessment (HK-MoCA) score equal to or less than 22; and (4) had a level of frailty from “managing well” to “living with severe frailty” (Clinical Frailty Scale score from 3 to 7) [33]. Participants were excluded if (1) they received any kind of rehabilitation service or (2) were living with another older adult who was participating in the same study.

For individuals deemed eligible to participate in the program, the research assistant provided a detailed explanation of the study and obtained their written consent at the community center. Baseline data were collected from these participants. Using the Research Randomizer software, the subjects were randomly assigned to either the intervention or control group based on the generated group assignments. The group assignments were securely sealed and opened sequentially by the principal investigator during the randomization process. To maintain a high level of double-blinding, participants were informed that the intervention aimed to promote psychological health but they were not informed about their specific group assignment (intervention or control). Furthermore, the research assistant responsible for data collection remained blind to the group allocation, whereas the providers, including the community center staff, were not blinded.

### Intervention Group

The 18-week intervention program contained 12 exercise sessions, which were supervised by occupational therapists and included the assistance of youth volunteers. Prior to commencing the intervention, youth volunteers attended educational training about the study at the community center. The youth volunteers were individuals between the ages of 17 and 35 years who were unemployed and had an educational level of secondary 5 or above. The training provided an overview of chronic disease and the biomechanical rationale of how dysfunction would interfere with performance in daily activities. The training also introduced the PEO model, an analytical tool, and established the concepts of interaction with older adults, application of digital technology and assistive devices, and environmental adaptations during the exercise protocol.

The intervention was an individualized exercise program using motion-based technology developed by occupational therapists (OTs). The first visit was conducted at the community centers so the OT could provide a comprehensive assessment of participants using a standardized assessment protocol. The assessment included the participant’s frailty level (eg, energy, physical ability, cognition, and health), capabilities and constraints, and environmental enablers and barriers. The results of the assessment and PEO model guided the OT to create a complete profile of the participant and construct an individualized exercise regime for the sequent follow-up phase. For example, the type of motion-based exercises prescribed could include video game–based activities focused on improving balance, such as virtual reality games that challenge participants to shift their weight and step in different directions. The intensity of these exercises would be tailored to the individual’s current fitness level and goals, such as building strength versus improving flexibility. The OT would also incorporate strategies to improve the participant’s engagement and meet their identified goals. This could include compensating for specific impairments by providing assistive technologies, like tablet stands or voice-controlled smart home devices, as well as advocating for environmental changes to minimize physical barriers in the home or community, such as installing grab bars or improving lighting.

Following the first meeting, 11 follow-up home visits were arranged with participants, including 5 weekly visits in the first month, and 6 biweekly visits in the next 3 months. The trained youth volunteers were asked to provide technical support to the older adults to help them get familiar with the digital interface and engage in the MBVGs prescribed. Meanwhile, volunteers also provided psychological support to help older adults cope with their fear of using technologies.

### Control Group

As with the participants in the intervention group, those in the control group could receive usual community center services such as health talks and physical activity class. They were also allowed to play any motion-based interactive games if they were interested, but without the guidance of youth volunteers or OTs.

### Sample Size

Sample size calculation was based on power analysis. Assuming a 2-tailed α of .05, a probability of .2 for β error (80% power), and an effect size of 0.45 after calculating with respect to the primary parameter (physical activities) from the result of a previous similar article [34], it was determined that 158 subjects were required. With an anticipated dropout rate of 20%, a total of 190 subjects were required (ie, 95 subjects per group).

### Data Collection

The data were collected at 2 time points: at baseline preintervention (T1) and at 18 weeks, when the program was completed (T2). The data were collected during home visits that were arranged before and after the program. Research assistants who were blinded to the grouping were responsible for data collection.

### Outcome Measures

#### Overview

There are 5 sets of measures, including demographics, physical frailty outcomes, cognitive frailty outcomes, social frailty outcomes, and client outcomes.

The demographic data—including age, gender, marital status, education, working and living conditions, accommodation, financial status, and caretaking support—were collected at T1.

#### Physical Frailty Outcomes (Balance, Handgrip Strength, Blood Pressure, BMI)

Balance was assessed using the Berg functional balance scale [35]. It is a 14-item scale designed to measure the balance of older adults in a community setting. Handgrip strength and blood pressure were measured using a calibrated hand dynamometer [36] and an electronic sphygmomanometer [37], respectively. BMI was calculated by dividing a subject’s weight in kilograms by his or her height in meters squared.

#### Cognitive Frailty Outcomes (Cognition, Short-Term Memory)

Cognition was assessed using the HK-MoCA. It has been shown to have consistency and reliability in detecting cognitive decline in an older adult population. MoCA covers the cognitive domains of short-term and working memory, visuospatial abilities, executive function, language, attention, concentration, and orientation. It has a maximum score of 30. The HK-MoCA cutoff score for mild Alzheimer disease is 18/19, different from the MoCA in English, which is 25/26 [38].

Short-term memory was assessed using the digit span forward test. It consists of the presentation of a list of numbers, which should be correctly repeated in a forward order immediately after their presentation. The longest span correctly recalled across all test items equals the highest number of repeated correct sequences. The tests have high test reliability coefficients (Fisher *z*=0.90) [39].

#### Social Frailty Outcomes (Loneliness, Social Isolation)

Loneliness was assessed using the University of California, Los Angeles Loneliness Scale [40]. Each participant was asked the following 3 questions: “How often do you feel that you lack companionship?” “How often do you feel left out?” and “How often do you feel isolated from others?” Each question had 3 options to reflect the frequency: 1=hardly ever, 2=some of the time, and 3=often. The values for each question were summed to get a loneliness score ranging from 3 to 9, with higher values indicating greater loneliness. The scale had good internal reliability in a previous study, with Cronbach α=.87 [41].

Social isolation was measured by the 2 subscales of the 6-item Lubben Social Network Scale-6 (LSNS-6) [42]. The LSNS-6 is composed of a set of 3 questions that evaluate social connectedness with relatives (LSNS-6 Family subscale) and a comparable set of 3 questions that evaluate social connectedness with friends (LSNS-6 Friends subscale). Specifically, the questions of the LSNS-6 Family subscale are as follows: “How many relatives do you see or hear from at least once a month?” “How many relatives do you feel close to such that you could call on them for help?” and “How many relatives do you feel at ease with that you can talk about private matters?” The word “relatives” in these 3 questions is replaced with the word “friends” for the questions of the LSNS-6 Friends subscale. Each subscale score ranges from 0 to 15, with a lower score indicating greater isolation. The 2 subscales in the previous study demonstrated good internal consistency reliability, with a Cronbach α of .81 for the Family subscale and .80 for the Friend subscale [41].

#### Client Outcomes (Physical Activities, Quality of Life, Depression, Self-Efficacy, Social Connectedness, eHealth Literacy)

Physical activities of the older adults were measured by the Chinese version of the Physical Activity Scale for the Elderly. It is a 12-item scale estimating the frequency and intensity of older adults’ lifestyle physical activities with 3 types of physical activities (leisure time activity: 5 items; household activity: 6 items; work-related activity: 1 item) during the previous 7-day period. The total score is computed by multiplying the time spent on each activity (recorded as never, seldom: 1‐2 days per week; sometimes: 3‐4 days per week; and often: 5‐7 days per week) or participation (yes/no) by an item’s weight, and summarizing all the items. The scale has a high test-retest reliability coefficient (*r*=0.87) and concurrent validity [43].

Quality of life was measured by a 12-item short-form health survey version 2 (SF-12v2), which has been translated, validated, and proven reliable for use among the Hong Kong Chinese population. The internal consistency and test-retest reliabilities were good (range 0.67‐0.82), and the SF-12v2 summary scores explained >80% of the total variances of the SF-36v2 summary scores [44].

Depression was measured with the Chinese version of the Geriatric Depression Scale [45]. Good validity and reliability were reported in this scale, with a criterion-related validity of 0.95 and test-retest reliability of 0.85 among Chinese older adults. Sensitivity and specificity were 96.3% and 87.5%, respectively, for a cutoff point of 8.

Self-efficacy was assessed using the Chinese version of the General Self-Efficacy Scale [46]. It is a 10-item scale measuring a broad and stable sense of personal competence to efficiently deal with a variety of stressful situations. The scale measures the strength dimension of self-efficacy on a 4-point Likert scale. Scores are summed to give a total range from 10 to 40; higher scores represent greater self-efficacy.

Social connectedness was assessed using the Social Connectedness Scale - Revised. Like its predecessor, this scale measures social connectedness as a psychological sense of belonging or, more specifically, as a cognition of enduring interpersonal closeness with the social world in toto. The scale consists of 20 items (10 positive and 10 negative) rated on a 6-point Likert scale and it has demonstrated good internal reliability [47].

Finally, eHealth literacy was measured using the Chinese version of the eHealth Literacy Scale [48]. This 8-item scale is used to measure an individual’s combined knowledge, comfort, and perceived skills related to finding, evaluating, and applying electronic tools to health problems. The scores of the scale range from 8 to 40, with higher scores indicating higher levels of eHealth literacy. The scale presented good reliability and validity [48].

### Data Analysis

The data were analysed using SPSS (version 29; IBM Corp). The participants’ baseline characteristics were compared using the chi-square test or Fisher exact test for categorical variables and the 2-sample independent *t* test for continuous variables. The *P* value was set at less than .05 as a significant result for the 2-tailed test. The between-group (group), within-group (time), and interaction effects (group × time) of outcome variables were analyzed using the generalized estimating equation, with Bonferroni adjustment to protect against the inflated risk of a type I error because of multiple comparisons [49]. The linear link function was used for all outcome measures. Missing values were imputed by multiple imputation after confirming the data were missing at random. We used the multiple imputation by chained equations approach, which generates multiple complete datasets by replacing missing values with predicted values derived from other variables in the dataset. Once the imputation was performed, we combined the results from the multiple datasets using Rubin’s rules. The primary analysis method used was the intention-to-treat approach, while the secondary analysis used the per-protocol method. No differences in the results were identified between the 2 analysis approaches.

### Ethical Considerations

Ethical approval was sought from the Ethics Committee of The Hong Kong Polytechnic University before the commencement of the program (reference number HSEARS20220225001). Information regarding procedures, risks, confidentiality, data storage, and benefits were provided to all eligible subjects. Written informed consent was obtained from the subjects. All participant names were replaced with participant codes to ensure confidentiality and anonymity. The digital data were stored in locked and secure computers. In order to ensure the safety of the study, the incidence of serious adverse events such as fainting and falls was monitored, though no harm to the subjects was found. Participants in this study received compensation for their time and participation, which covered expsenses such as travel costs. The compensation details were transparently outlined during the informed consent process.

## Results

### Baseline Demographic Data

**Figure 1.**
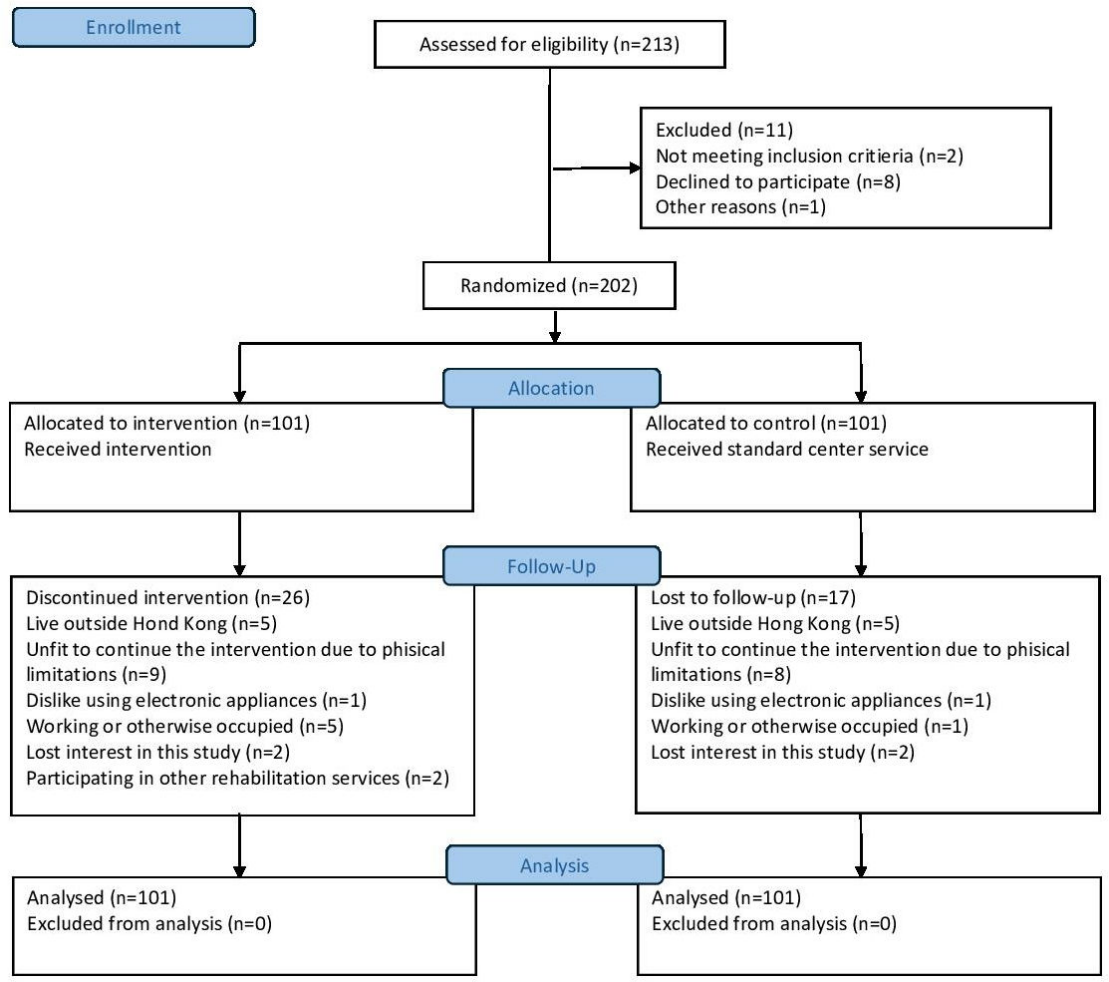
The CONSORT flow diagram. CONSORT: Consolidated Standards of Reporting Trials.

A total of 202 participants from 6 community centers were recruited and randomly assigned to either the intervention group (n=101) or the control group (n=101). During the program, 26 participants from the intervention group and 17 participants from the control group dropped out for various reasons, such as a move to another country (n=10), deterioration of the participant’s physical condition (n=17), and a dislike of using electronic appliances (n=2). The CONSORT (Consolidated Standards of Reporting Trials) flow diagram can be found in Figure 1.

Baseline demographic characteristics were balanced across the 2 groups (Multimedia Appendix 1). The mean age of the 202 participants was 78.8 years (SD 7.8) and only 21.8% (n=44) had no formal education. All but 6 were retired. More than half of the participants were living with their spouse or family (n=132, 65.3%). The majority (n=183, 90.6%) indicated having adequate or more than adequate financial resources. Most of them said that they are able to take care of themselves (n=145, 71.8%). Some of them said that they are being taken care of by their children (n=126, 62.4%) and spouse (n=40, 19.8%).

### Outcomes

Both groups demonstrated significant improvements in balance from T1 to T2, with a notable time effect (*β*=−0.63, 95% CI −1.22 to −0.05; *P*=.03). Although the intervention group showed improvements in right- and left-hand strength compared to the control group, the differences were not statistically significant. Similarly, no significant differences were found between groups for systolic and diastolic blood pressure, short-term memory, social isolation, physical activities, or quality of life components.

The intervention group exhibited D improvements in BMI (interaction effect at T1: *β*=−1.00, 95% CI −1.91 to −0.08; *P*=.03) and cognition (interaction effect at T1: *β*=2.43, 95% CI 1.08 to 3.78; *P*<.001), while the control group’s MoCA scores decreased. Both groups improved in depression levels, with a significant within-group effect at T2 for the intervention group (*β*=−1.16, 95% CI −1.85 to −0.48; *P*=.001).

Furthermore, eHealth literacy also improved in both groups, with a significant within-group effect at T2 for the intervention group (*β*=3.56, 95% CI 1.33 to 5.78; *P*=.002). Despite these improvements, no significant between-group differences were observed across most measures, highlighting the lack of significant interaction effects.

**Figure 2.**
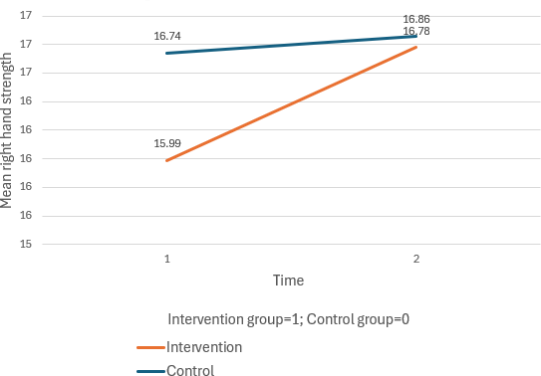
Mean changes in right hand strength across time for the intervention and control group.

**Figure 3.**
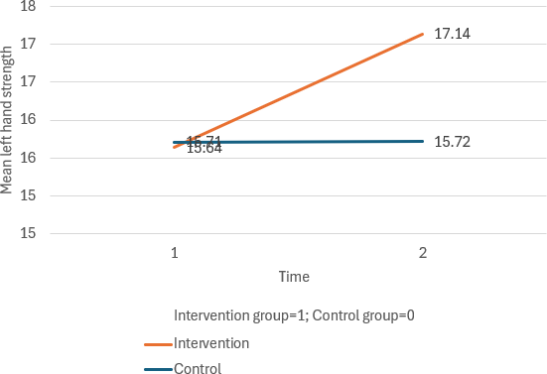
Mean changes in left hand strength across time for the intervention and control group.

**Figure 4.**
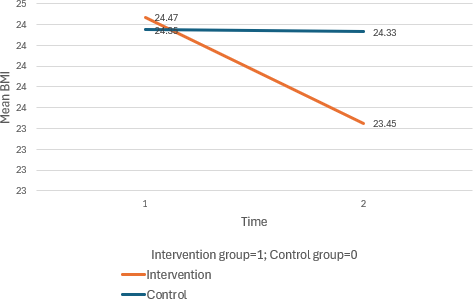
Mean changes in BMI across time for the intervention and control group.

**Figure 5.**
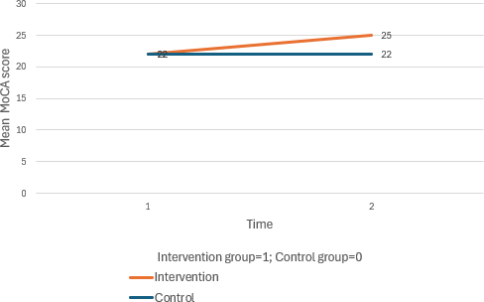
Mean changes in total MoCA score across time for the intervention and control group. MoCA: Montreal Cognitive Assessment.

**Figure 6.**
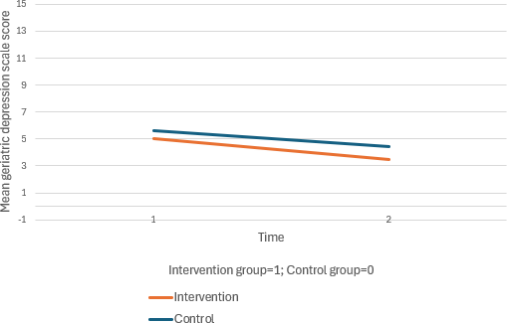
Mean changes in total geriatric depression scale score across time for the intervention and control group.

**Figure 7.**
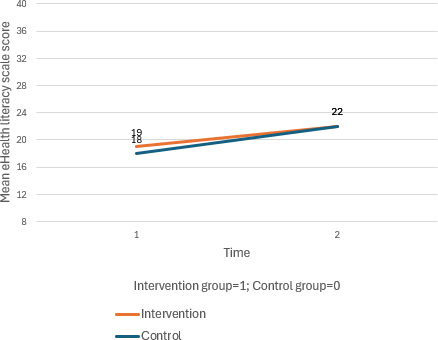
Mean changes in eHealth literacy scale score across time for the intervention and control group.

The full results are presented in Tables1  2 and Figures 2-7.

**Table 1.** Mean scores and values for balance, handgrip strength, blood pressure, BMI, cognition, short-term memory, loneliness, social isolation, physical activities, quality of life, depression, self-efficacy, social connectedness, and eHealth literacy among frail community-dwelling older adults for the intervention and control group at baseline (T1) and 18 weeks (T2).

Outcomes and groups	Mean (SE)	95% Wald CI
**Balance**		
***Control group***		
T2	4.99 (0.28)	4.45-5.53
T1	5.62 (0.27)	5.1-6.14
***Intervention group***		
T2	5.25 (0.3)	4.67-5.84
T1	5.75 (0.29)	5.19-6.31
**Right hand strength**		
***Control group***		
T2	16.86 (0.75)	15.38-18.34
T1	16.74 (0.71)	15.35-18.13
*** Intervention group***		
T2	16.78 (0.9)	15.01-18.54
T1	15.99 (0.74)	14.54-17.44
**Left hand strength**		
***Control group***		
T2	15.72 (0.69)	14.38-17.07
T1	15.71 (0.68)	14.37-17.06
***Intervention group***		
T2	17.14 (0.8)	15.56-18.71
T1	15.64 (0.68)	14.31-16.97
**Systolic blood pressure**		
***Control group***		
T2	135.42 (2.143)	131.22-139.62
T1	137.91 (2.104)	133.79-142.03
***Intervention group***		
T2	132.13 (2.308)	127.61-136.66
T1	135.86 (1.840)	132.26-139.47
**Diastolic blood pressure**		
***Control group***		
T2	72.24 (1.616)	69.07-75.40
T1	74.02 (1.011)	72.04-76.00
***Intervention group***		
T2	73.45 (1.138)	71.22-75.68
T1	74.00 (1.008)	72.02-75.98
**BMI**		
***Control group***		
T2	24.33 (0.52)	23.32-25.34
T1	24.35 (0.46)	23.44-25.26
***Intervention group***		
T2	23.45 (0.42)	22.63-24.27
T1	24.47 (0.38)	23.74-25.21
**Cognition**		
***Control group***		
T2	21.95 (0.642)	20.69-23.21
T1	22.21 (0.534)	21.16-23.25
***Intervention group***		
T2	24.72 (0.37)	23.99-25.45
T1	22.54 (0.49)	21.59-23.50
**Short-term memory**		
***Control group***		
T2	11.74 (0.263)	11.22-12.25
T1	11.89 (0.294)	11.31-12.47
*** Intervention group***		
T2	11.96 (0.268)	11.44-12.48
T1	12.18 (0.296)	11.60-12.76
**Loneliness**		
***Control group***		
T2	4.39 (0.17)	4.06-4.71
T1	4.07 (0.14)	3.80-4.34
*** Intervention group***		
T2	4.47 (0.18)	4.12-4.81
T1	4.01 (0.14)	3.74-4.28
**Social isolation**		
***Control group***		
T2	11.54 (0.70)	10.17-12.91
T1	10.37 (0.56)	9.27-11.47
***Intervention group***		
T2	10.30 (0.62)	9.08-11.52
T1	10.42 (0.65)	9.14-11.69
**Physical activities**		
***Control group***		
T2	77.00 (5.00)	67.21-86.80
T1	83.95 (4.66)	74.82-93.08
***Intervention group***		
T2	95.35 (7.38)	80.89-109.80
T1	91.67 (5.57)	80.75-102.59
**Quality of life (physical component)**		
***Control group***		
T2	38.36 (1.19)	36.02-40.69
T1	37.13 (1.15)	34.87-39.38
*** Intervention group***		
T2	40.86 (0.98)	38.94-42.78
T1	38.73 (0.94)	36.89-40.58
**Quality of life (mental component)**		
***Control group***		
T2	44.75 (1.02)	42.71-46.79
T1	44.72 (1.01)	42.69-46.75
***Intervention group***		
T2	44.34 (1.28)	41.83-46.84
T1	48.30 (1.17)	46.00-50.60
**Depression**		
***Control group***		
T2	4.42 (0.41)	3.62-5.23
T1	5.58 (0.38)	4.84-6.33
***Intervention group***		
T2	3.55 (0.37)	2.81-4.28
T1	5.05 (0.35)	4.36-5.74
**Self-efficacy**		
***Control group***		
T2	25.51 (0.77)	24.00-27.02
T1	23.88 (0.68)	22.54-25.22
***Intervention group***		
T2	25.33 (0.64)	24.08-26.59
T1	25.17 (0.66)	23.88-26.46
**Social connectedness**		
***Control group***		
T2	78.96 (1.63)	75.77-82.15
T1	80.92 (1.42)	78.13-83.71
***Intervention group***		
T2	75.55 (1.44)	72.73-78.37
T1	81.60 (1.42)	78.82-84.39
**eHealth literacy**		
***Control group***		
T2	21.41 (0.92)	19.61-23.21
T1	17.85 (0.99)	15.91-19.79
*** Intervention group***		
T2	21.61 (0.88)	19.90-23.33
T1	19.00 (0.94)	17.16-20.84

**Table 2.** The between-group (group), within-group (time), and interaction effects (group × time) of balance, handgrip strength, blood pressure, BMI, cognition, short-term memory, loneliness, social isolation, physical activities, quality of life, depression, self-efficacy, social connectedness, and eHealth literacy.

		β (SE)	95% CI	Wald chi-square (df)	*P* value
**Balance**					
	Intercept	5.624 (0.2656)	5.103 to 6.144	448.274	<.001^a^
	Intervention group	0.129 (0.3896)	−0.635 to 0.892	0.109	.74
	Time=2	−0.636 (0.2970)	−1.218 to −0.054	4.582	.03^a^
	Interaction of intervention group and time	0.137 (0.4457)	−0.737 to 1.010	0.094	.76
**Right hand strength**					
	Intercept	16.740 (0.7101)	15.348 to 18.132	555.729	<.001^a^
	Intervention group	−0.752 (1.0255)	−2.762 to 1.258	0.538	.46
	Time=2	0.123 (0.8026)	−1.451 to 1.696	0.023	.88
	Interaction of intervention group and time	0.666 (1.3347)	−1.950 to 3.282	0.249	.62
**Left hand strength**					
	Intercept	15.713 (0.6850)	14.371 to 17.056	526.261	<.001^a^
	Intervention group	−0.071 (0.9636)	−1.960 to 1.817	0.005	.94
	Time=2	0.010 (0.7316)	−1.424 to 1.444	0.000	.99
	Interaction of intervention group and time	1.486 (1.1884)	−0.843 to 3.815	1.563	.21
**Systolic blood pressure**					
	Intercept	137.911 (2.1036)	133.788 to 142.034	4297.909	<.001^a^
	Intervention group	−2.050 (2.7948)	−7.527 to 3.428	0.538	.46
	Time=2	−2.494 (2.2081)	−6.822 to 1.834	1.276	.26
	Interaction of intervention group and time	−1.234 (3.3668)	−7.833 to 5.365	0.134	.71
**Diastolic blood pressure**					
	Intercept	74.020 (1.0109)	72.039 to 76.001	5361.741	<.001^a^
	Intervention group	−0.020 (1.4277)	−2.818 to 2.778	0.000	.99
	Time=2	−1.782 (1.6507)	−5.017 to 1.454	1.165	.28
	Interaction of intervention group and time	1.235 (2.0235)	−2.731 to 5.201	0.373	.54
**BMI**					
	Intercept	24.354 (0.4645)	23.443 to 25.264	2748.915	<.001^a^
	Intervention group	0.118 (0.5974)	−1.053 to 1.288	0.039	.84
	Time=2	−0.025 (0.2885)	−0.590 to 0.540	0.007	.93
	Interaction of intervention group and time	−0.995 (0.4651)	−1.906 to −0.083	4.573	.03^a^
**Cognition**					
	Intercept	22.208 (0.5340)	21.161 to 23.254	1729.695	<.001^a^
	Intervention group	0.337 (0.7227)	−1.080 to 1.753	0.217	.64
	Time=2	−0.256 (0.4970)	−1.230 to 0.719	0.264	.61
	Interaction of intervention group and time	2.431 (0.6902)	1.078 to 3.784	12.406	<.001^a^
**Short-term memory**					
	Intercept	11.891 (0.2943)	11.314 to 12.468	1632.041	<.001^a^
	Intervention group	0.287 (0.4176)	−0.531 to 1.106	0.473	.49
	Time=2	−0.153 (0.3082)	−0.757 to 0.451	0.246	.62
	Interaction of intervention group and time	−0.065 (0.4315)	−0.911 to 0.781	0.023	.88
**Loneliness**					
	Intercept	4.069 (0.1367)	3.801 to 4.337	886.655	<.001^a^
	Intervention group	−0.059 (0.1954)	−0.442 to 0.324	0.092	.76
	Time=2	.316 (0.1825)	−0.041 to 0.674	3.003	.08
	Interaction of intervention group and time	0.141 (0.2671)	−0.383 to 0.664	0.277	.60
**Social isolation**					
	Intercept	10.366 (0.5607)	9.267 to 11.465	341.770	<.001^a^
	Intervention group	0.050 (0.8596)	−1.635 to 1.734	0.003	.95
	Time=2	1.170 (0.7479)	−0.296 to 2.636	2.449	.12
	Interaction of intervention group and time	−1.289 (1.0510)	−3.349 to 0.771	1.504	.22
**Physical activities**					
	Intercept	83.948 (4.6577)	74.819 to 93.077	324.850	<.001^a^
	Intervention group	7.723 (7.2628)	−6.512 to 21.958	1.131	.29
	Time=2	−6.944 (4.8357)	−16.422 to 2.534	2.062	.15
	Interaction of intervention group and time	10.620 (9.1539)	−7.322 to 28.561	1.346	.25
**Quality of life (physical component)**					
	Intercept	37.126 (1.1494)	34.873 to 39.379	1043.212	<.001^a^
	Intervention group	1.608 (1.4864)	−1.306 to 4.521	1.170	.28
	Time=2	1.234 (1.2172)	−1.151 to 3.620	1.028	.31
	Interaction of intervention group and time	0.894 (1.6410)	−2.323 to 4.110	0.296	.59
**Quality of life (mental component)**					
	Intercept	49.402 (1.0957)	47.255 to 51.550	2032.807	<.001^a^
	Intervention group	−1.099 (1.6054)	−4.245 to 2.048	0.468	.49
	Time=2	−0.477 (1.1842)	−2.798 to 1.844	0.162	.69
	Interaction of intervention group and time	−3.489 (1.9421)	−7.296 to 0.317	3.227	.07
**Depression**					
	Intercept	5.584 (0.3817)	4.836 to 6.332	213.985	<.001^a^
	Intervention group	−0.535 (0.5197)	−1.553 to 0.484	1.058	.30
	Time=2	−1.162 (0.3503)	−1.849 to −0.476	11.012	.001^a^
	Interaction of intervention group and time	−0.340 (0.5236)	−1.367 to 0.686	0.423	.52
**Self-efficacy**					
	Intercept	23.881 (0.6826)	22.543 to 25.219	1224.125	<.001^a^
	Intervention group	1.287 (0.9473)	−0.570 to 3.144	1.846	.17
	Time=2	1.631 (0.8629)	−0.060 to 3.322	3.572	.06
	Interaction of intervention group and time	−1.466 (1.1811)	−3.781 to 0.849	1.541	.22
**Social connectedness**					
	Intercept	80.921 (1.4217)	78.134 to 83.707	3239.572	<.001^a^
	Intervention group	0.683 (2.0106)	−3.258 to 4.624	0.115	.73
	Time=2	−1.957 (1.6042)	−5.102 to 1.187	1.489	.22
	Interaction of intervention group and time	−4.100 (2.3944)	−8.793 to 0.593	2.932	.09
**eHealth literacy**					
	Intercept	17.851 (0.9909)	15.909 to 19.794	324.537	<.001^a^
	Intervention group	1.149 (1.3658)	−1.528 to 3.825	0.707	.40
	Time=2	3.558 (1.1351)	1.333 to 5.783	9.826	.002^a^
	Interaction of intervention group and time	−0.945 (1.5229)	−3.930 to 2.040	0.385	.54

a *P*<.05.

## Discussion

### Principal Findings

The increasingly aging population across the world warrants the development and implementation of novel strategies to promote aging in place. In this study, we examined the effects of individualized exercise programs using a combination of frames of reference, intergenerational support, and MBVGs. Overall, there were statistically nonsignificant results across most outcomes except for BMI and cognition. Although improved mean scores were observed for some outcomes such as loneliness, balance, eHealth literacy, and social isolation, these were not statistically significant across the groups. Although the findings highlight the limited benefits associated with the novel, comprehensive program implemented in this study, this program may represent a foundation for further work to improve biopsychosocial outcomes among older adults and to promote aging in place for as long as practicable.

Video-based gaming interventions have generally been observed to be potentially helpful at improving cognitive functions and physical outcomes among older adults [50]. In a recent meta-analysis that included 47 studies, it was reported that video game interventions could be considered for older adults to improve performance and cognitive function, especially general cognitive scores and processing speed [51]. For older adults in residential care, it has been reported that MBVGs are helpful for both mental and physical stimulation [52]. Consistent with these assertions, this study observed an improvement in cognition. This finding may be related to the nature of the game, which requires one to think through the game, ascertain the next steps to take in the game, and work toward winning. MBVGs can facilitate the development and improvement of spatial awareness, attention span, spatial constraints, and executive control skills [51,53]. All these can potentially contribute to improving cognitive function [54].

Further to the above, we observed a statistical improvement in BMI levels in this study, although the findings regarding physical activity were not statistically significant. Potentially, the movement required to play the game may have contributed to marginal weight reduction over the study period and led to changes in BMI. However, the negative finding observed regarding physical activity may suggest that the physical actions required to play the games may not necessarily translate to participating in other physical activities. There was ongoing support offered to participants during each episode of the gaming intervention, whereas in reality, there may be limited support to enable them to participate in other physical activities. Activity levels vary across the older adult population, although this was not taken into consideration in this study. Moving forward, this finding may underscore a need to ascertain the activity level of participants, which can help to attain a greater explanatory power regarding the changes that occur over time.

Apart from cognition and BMI, all remaining biopsychosocial outcomes were statistically insignificant. Although it remains a rather challenging endeavor to ascertain why this was the case, particularly considering the comprehensive and individualized nature of the gaming intervention, it is possible that the added aspect of volunteers was of limited impact in this study. This is based on the assertion that participants in the control group were allowed to play the games if they wanted to, but without the support of community volunteers. Unlike older adults in residential facilities, community-dwelling older adults may still be able to do more for themselves, as they may have varying degrees of functional limitations [55]. Thus, they may not necessarily require extensive, ongoing support as offered by the intervention. Instead, this form of ongoing support should be offered on a case-by-case basis to community-dwelling older adults who may be in need of such support. Another potential explanation regarding the nonsignificant findings may perhaps be related to the intergenerational gap between the community-dwelling older adults and the younger volunteers; they may have had varying worldviews and this could have impacted the social interactions that emerged from such relationships, affecting the support offered or received. This influence may impede social bonding with the younger volunteers and hinder older adults’ active participation in social connectedness and engagement, thereby potentially affecting outcomes related to social well-being. Additionally, the older adult population is heterogeneous, with varying underlying comorbidities, which was not taken into consideration in this study. The pathological basis of underlying comorbidities such as diabetes and hypertension can often impact psychosocial outcomes. A more homogeneous group of community-dwelling adults may offer results allowing stronger comparisons.

### Future Implications

Future research may consider whether the intervention was tailored enough to address the varied requirements of older adults with frailty. Subsequent research could investigate more customized methods to effectively cater to individual abilities and preferences. The add-on aspect of volunteer support may be considered on a case-by-case basis rather than a one-size-fit-all approach. In addition, other specific components of the MBVGs, such as exercise type, intensity, and duration, could be systematically evaluated and adjusted to potentially enhance the efficacy of the intervention. Future studies could further examine individual differences to determine if specific demographic groups show different levels of interest or enjoyment when engaging with specific components of MBVGs. These subtleties can offer more insights into how game design can be adjusted to better cater to the diverse preferences and requirements of players.

### Limitations

Some limitations of the study should be noted. First, due to the selection of participants based on their interests, this study exclusively recruited individuals who were highly motivated and proactive in the intervention, potentially leading to recruitment bias and limiting sample diversity. Second, it should be acknowledged that the community centers were unable to assign the same youth volunteer to each participant throughout the program. This was due to certain volunteers being relocated to another country during the period or leaving the program due to a lack of interest or time constraints. The presence of different supporters each time may have resulted in less relationship-building between the youth volunteers and the older adults, potentially impacting the effectiveness of the program. Third, there were multiple outcome measures that required approximately 1 hour for older participants to complete. The potential fatigue experienced by participants during these assessments could have a negative impact on the reliability of the results. Fourth, the COVID-19 pandemic necessitated a shift in our training delivery method from face-to-face to Zoom. This change in mode of instruction may have affected the quality of teaching, particularly when it came to demonstrating motion-based games. Finally, no follow-up assessment was conducted, as the study was a preliminary investigation laying the foundation for future research. Subsequent studies may incorporate additional follow-up assessments to evaluate the sustained long-term progress.

### Conclusion

Undoubtedly, the growing aging population warrants the development and implementation of creative strategies to support them. A motion-based gaming intervention with the add-on aspect of younger volunteers for support seemed to confer limited benefits to older adults.

## Supplementary material

10.2196/57352 Multimedia Appendix 1 Demographic characteristics of participants.

10.2196/57352 Checklist 1 CONSORT-EHEALTH checklist (V 1.6.1).

## Abbreviations

CONSORT: Consolidated Standards of Reporting Trials
HK-MoCA: Hong Kong version of the Montreal Cognitive Assessment
LSNS-6: 6-item Lubben Social Network Scale-6
MBVG: motion-based video game
MoCA: Montreal Cognitive Assessment
OT: occupational therapist
PEO: Person-Environment-Occupation
SF-12v2: 12-item short-form health survey version 2
