# Supplementary material for: The Effect of Young People–Assisted, Individualized, Motion-Based Video Games on Physical, Cognitive, and Social Frailty Among Community-Dwelling Older Adults With Frailty: Randomized Controlled Trial
Source: JMIR Serious Games. 2024 Nov 20;12:e57352. doi: 10.2196/57352 (PMC11600656; doi:10.2196/57352)
Supplement: Multimedia Appendix 1 [file games-v12-e57352-s001.docx]

Appendix 1. Demographic characteristics of participants (N=202)

|  |  | **Total (N=202)** |  | **Intervention Group (n=101)** |  | **Control Group (n=101)** |  |  |
| --- | --- | --- | --- | --- | --- | --- | --- | --- |
|  |  | N | % | n | % | n | % | *P*-value Chi-square test/ Fisher's exact test |
| **Gender** | Male | 31 | 15.3% | 18 | 17.8% | 13 | 12.9% | 0.33 |
|  | Female | 171 | 84.7% | 83 | 82.2% | 88 | 87.1% |  |
| **Age** | Means (SD) | 78.80 (7.76) | NA | 78.41 (8.18) | NA | 79.17 (7.33) | NA | 0.48 |
|  | Median (range) | 78.5 (60-95) | NA | 79 (60-95) | NA | 78 (64-93) | NA |  |
| **Marital Status** | Single | 4 | 2.0% | 3 | 3.0% | 1 | 1.0% | 0.36 |
|  | Married | 70 | 34.7% | 37 | 36.6% | 33 | 32.7% |  |
|  | Divorced | 14 | 6.9% | 9 | 8.9% | 5 | 5.0% |  |
|  | Widowed | 114 | 56.4% | 52 | 51.5% | 62 | 61.4% |  |
| **Education Level** | No formal education | 44 | 21.8% | 17 | 16.8% | 27 | 26.7% | 0.56 |
|  | Primary school | 93 | 46.0% | 49 | 48.5% | 44 | 43.6% |  |
|  | Secondary school | 56 | 27.7% | 30 | 29.7% | 26 | 25.7% |  |
|  | Tertiary or above | 7 | 3.5% | 4 | 4.0% | 3 | 3.0% |  |
| **Working condition** | Retired | 196 | 97.0% | 98 | 97.0% | 98 | 97.0% | 0.72 |
|  | Full-time | 3 | 1.5% | 2 | 2.0% | 1 | 1.0% |  |
|  | Part-time | 3 | 1.5% | 1 | 1.0% | 2 | 2.0% |  |
| **Living Conditions** | Flat | 192 | 95.0% | 95 | 94.1% | 97 | 96.0% | 0.52 |
|  | Sub-divided flat | 10 | 5.0% | 6 | 5.9% | 4 | 4.0% |  |
| **Living** | Alone | 70 | 34.7% | 31 | 30.7% | 39 | 38.6% | 0.40 |
|  | With spouse | 39 | 19.3% | 19 | 18.8% | 20 | 19.8% |  |
|  | With family | 93 | 46.0% | 51 | 50.5% | 42 | 41.6% |  |
| **Financial Status** | More than adequate | 47 | 23.3% | 20 | 19.8% | 27 | 26.7% | 0.46 |
|  | Adequate | 136 | 67.3% | 72 | 71.3% | 64 | 63.4% |  |
|  | Inadequate | 19 | 9.4% | 9 | 8.9% | 10 | 9.9% |  |
|  | Very inadequate | 0 | 0.0% | 0 | 0.0% | 0 | 0.0% |  |
| **Caregiver: Self** | Yes | 145 | 71.8% | 68 | 67.3% | 77 | 76.2% | 0.16 |
|  | No | 57 | 28.2% | 33 | 32.7% | 24 | 23.8% |  |
| **Caregiver: Spouse** | Yes | 40 | 19.8% | 21 | 20.8% | 19 | 18.8% | 0.72 |
|  | No | 162 | 80.2% | 80 | 79.2% | 82 | 81.2% |  |
| **Caregiver: Siblings** | Yes | 5 | 2.5% | 2 | 2.0% | 3 | 3.0% | 1.00 |
|  | No | 197 | 97.5% | 99 | 98.0% | 98 | 97.0% |  |
| **Caregiver: Children** | Yes | 126 | 62.4% | 69 | 68.3% | 57 | 56.4% | 0.08 |
|  | No | 76 | 37.6% | 32 | 31.7% | 44 | 43.6% |  |
| **Caregiver: Children-in-law** | Yes | 10 | 5.0% | 6 | 5.9% | 4 | 4.0% | 0.52 |
|  | No | 192 | 95.0% | 95 | 94.1% | 97 | 96.0% |  |
| **Caregiver: Friends** | Yes | 5 | 2.5% | 3 | 3.0% | 2 | 2.0% | 1.00 |
|  | No | 197 | 97.5% | 98 | 97.0% | 99 | 98.0% |  |
| **Caregiver: Neighbors** | Yes | 3 | 1.5% | 2 | 2.0% | 1 | 1.0% | 1.00 |
|  | No | 199 | 98.5% | 99 | 98.0% | 100 | 99.0% |  |
| **Caregiver: Volunteers** | Yes | 5 | 2.5% | 1 | 1.0% | 4 | 4.0% | 0.37 |
|  | No | 197 | 97.5% | 100 | 99.0% | 97 | 96.0% |  |
| **Caregiver: Domestic helpers** | Yes | 25 | 12.4% | 12 | 11.9% | 13 | 12.9% | 0.83 |
|  | No | 177 | 87.6% | 89 | 88.1% | 88 | 87.1% |  |
| **Frequency of care** | Always | 105 | 52.0% | 57 | 56.4% | 48 | 47.5% | 0.51 |
|  | Sometimes | 49 | 24.3% | 23 | 22.8% | 26 | 25.7% |  |
|  | Only at night | 5 | 2.5% | 3 | 3.0% | 2 | 2.0% |  |
|  | No help from others | 43 | 21.3% | 18 | 17.8% | 25 | 24.8% |  |

*Note:* SD = Standard deviation **P* < .05 statistically significant
